# Supplementary material for: Association of abnormal pulmonary vasculature on CT scan for COVID-19 infection with decreased diffusion capacity in follow up: A retrospective cohort study
Source: PLoS One. 2021 Oct 15;16(10):e0257892. doi: 10.1371/journal.pone.0257892 (PMC8519442; doi:10.1371/journal.pone.0257892)
Supplement: Attachment — Submitted filename: Response to Reviewers.docx [file pone.0257892.s002.docx]

September 1^st^, 2021

RE: Association of Abnormal Pulmonary Vasculature on CT Scan for COVID-19 Infection with Decreased Diffusion Capacity in Follow Up: a retrospective cohort study.

Dear Editor:

Thank you for your consideration and feedback of our manuscript “Association of Abnormal Pulmonary Vasculature on CT Scan for COVID-19 Infection with Decreased Diffusion Capacity in Follow Up: a retrospective cohort study.”

1) Response to Academic Editor:

Thank you for your insightful review of our manuscript. We agree that the sample size is small and it would be difficult to extrapolate the numbers needed to analyze in order to make a more definitive conclusion about abnormal pulmonary vasculature and risk or developing a reduced DLCO. Could you elaborate? Would you like us to make a more definitive statement about the statistical power in the manuscript? Additionally, we would like you to elaborate on the statement “all factors should be considered in the multivariable analysis.” The following variables were used as covariates: age, sex, race, smoking status, lung volumes, and hemoglobin. In order to address this statement, could you be more specific?

2) Response to Reviewer #1:

Thank you for your insightful review of our manuscript. We completely agree with your assessment. We added in our limitations the following to reflect into your comments: *Sixth, we did not have in our cohort other test (such as ventilation-perfusions scans, CT pulmonary angiography, dual energy CT) that could have provided more insight into pulmonary vascular injury related to COVID-19 pulmonary disease.*

We also did some minor updates in the background and discussion section, we added 3 new references. We updated current number of COVID 19 cases and deaths in the US in the background section.

3) Funding Statement:

Image analyses were funded by Fluidda’s COVID19 consortium. The funders had no

role in study design, data collection and analysis, decision to publish, or preparation of

the manuscript.

4) Competing Interests:

Over the past three years, VK reports personal fees from Gala Therapeutics, the ABIM,

AstraZeneca, and Boehringer Ingelheim outside the submitted work; ML, JDB, BL have

received monies from Fluidda, Inc; IO, MD, RG, FJ, MEVS, DS have no competing

interests to declare.

5) Content Permission Form:

We are sending as a PDF the completed Content Permission Form for our Figure 2.

Thank you once again. Please contact me with any questions or concerns.

Sincerely,

Daniel Salerno MD, MS

Associate Professor of Medicine

Department of Thoracic Medicine and Surgery

Lewis Katz School of Medicine at Temple University

3401 North Broad Street

785 Parkinson Pavilion

Philadelphia, PA 19140

(215) 707-9929 (office)

(215)707-6867(fax)
